# Bioactive Lipids of Seaweeds from the Portuguese North Coast: Health Benefits versus Potential Contamination

**DOI:** 10.3390/foods10061366

**Published:** 2021-06-12

**Authors:** Cristina Soares, Sara Sousa, Susana Machado, Elsa Vieira, Ana P. Carvalho, Maria João Ramalhosa, Simone Morais, Manuela Correia, Teresa Oliva-Teles, Valentina F. Domingues, Cristina Delerue-Matos

**Affiliations:** 1 REQUIMTE/LAQV, Instituto Superior de Engenharia do Porto (ISEP), Instituto Politécnico do Porto, R. Dr. António Bernardino de Almeida 431, 4200-072 Porto, Portugal; cmdss@isep.ipp.pt (C.S.); sara_sousa_23@hotmail.com (S.S.); su_tche@hotmail.com (S.M.); elsa.vieiraf@graq.isep.ipp.pt (E.V.); apcarvalho@porto.ucp.pt (A.P.C.); mjr@isep.ipp.pt (M.J.R.); sbm@isep.ipp.pt (S.M.); mmb@isep.ipp.pt (M.C.); mtt@isep.ipp.pt (T.O.-T.); cmm@isep.ipp.pt (C.D.-M.); 2 REQUIMTE/LAQV, Department of Chemical Sciences, Faculty of Pharmacy, University of Porto, Rua Jorge Viterbo Ferreira, 228, 4050-313 Porto, Portugal; 3 CBQF—Centro de Biotecnologia e Química Fina—Laboratório Associado, Escola Superior de Biotecnologia, Universidade Católica Portuguesa, Rua Diogo Botelho 1327, 4169-005 Porto, Portugal

**Keywords:** aliphatic hydrocarbons, fatty acids, lipid profile, nutritional indices, polycyclic aromatic hydrocarbons, seaweeds nutrition

## Abstract

The total lipid content and lipidic profile of seaweeds harvested in the North Coast and purchased in Portugal were determined in this paper. The amount of total lipids in the different species of seaweeds varied between 0.7 ± 0.1% (*Chondrus crispus*) and 3.8 ± 0.6% (*Ulva* spp.). Regarding the fatty acid content, polyunsaturated fatty acids (PUFA) ranged between 0–35%, with *Ulva* spp. presenting the highest amount; monounsaturated fatty acids (MUFA) varied between 19 and 67%; and saturated fatty acids (SFA) were predominant in *C. crispus* (45–78%) and *Gracilaria* spp. (36–79%). Concerning the nutritional indices, the atherogenicity index (AI) was between 0.4–3.2, the thrombogenicity index (TI) ranged from 0.04 to 1.95, except for *Gracilaria* spp., which had a TI of 7.6, and the hypocholesterolemic/hypercholesterolemic ratio (HH) values ranged between 0.88–4.21, except for *Gracilaria* spp., which exhibited values between 0.22–9.26. The n6/n3 ratio was below 1 for most of the species evaluated, except for *Ascophyllum nodosum*, which presented a higher value, although below 2. Considering the PUFA/SFA ratio, seaweeds presented values between 0.11–1.02. The polycyclic aromatic hydrocarbons (PAHs) and aliphatic hydrocarbons (AHCs) contamination of seaweeds under study was also quantified, the values found being much lower than the maximum levels recommended for foodstuff.

## 1. Introduction

Seaweeds have been consumed worldwide since primordial times [1]. They are rich in proteins, fibers, minerals, vitamins, essential amino acids, and polyunsaturated fatty acids (PUFA). In addition to their nutritional contribution, these nutrients present a functional behavior, reducing the occurrence of certain chronic diseases, such as diabetes, obesity, heart disease, and cancer [2]. Seaweeds are widely used as food in Asian countries and to a lesser extent in Europe and America. Many seaweeds species are used in the food industry, and pharmaceutical applications are under investigation [3].

However, the exploration of natural resources by intensive wild harvesting has clear adverse environmental impacts, as the marine environment is vulnerable and dependent on a harmonious life equilibrium. Therefore, knowing the potential of marine life along with the development of sustainable systems for their farming, processing, and application are necessary to achieve a sustainable utilization of marine resources [4].

PUFA are well recognized in human nutrition for their essential role in health maintenance. Since the human body cannot produce linoleic (18:2 n-6) and alpha-linolenic (18:3 n-3) acids (essential fatty acids), their supply via food sources is imperative. However, other PUFA such as the long-chain eicosapentaenoic (EPA, 20:5 n-3) and docosahexaenoic (DHA, 22:6 n-3) acids are not classified as essential, but are also critical for normal development due to the strong knowledge linking their internal concentrations and several diseases, such as cardiovascular diseases, mental disorders, and inflammation, among others [5].

The usual dietary sources of PUFA, especially the long-chain PUFA, are marine sources, namely oily fish (e.g., mackerel, herring, sardine, and salmon). However, the worldwide depletion of fish populations leads to an urgent demand for alternative sources of these PUFA [6]. Despite its interesting PUFA content, fish itself cannot produce EPA and DHA, obtained from its dietary sources. In fact, in the marine environment, microalgae are the primary source of PUFA, which feeds the species in the upper levels of the food chain. Seaweeds usually present lower levels of PUFA than microalgae (phytoplankton), as they can be harvested and consumed in an easier way than the latter, as their production does not require expensive bioreactors and cell harvesting processes [7]. Therefore, they are an interesting alternative to microalgae as sources of healthy lipid products.

On the other hand, due to their ability to absorb and adsorb compounds, seaweeds also accumulate contaminants [1]. Consequently, they must be carefully assessed for safety reasons before used as food products [8,9]. Hydrocarbons are categorized as high-risk pollutants and high levels of contamination have been detected in different environmental matrices [10]. Aliphatic hydrocarbons (AHCs) correspond to linear and saturated carbon chains of C6-C40 from natural and anthropogenic AHCs sources, mainly originating from crude oil and liposoluble contaminants [10,11,12]. They are present in very diverse concentrations in almost all foods: dietary exposure to AHCs varied in the range of approximately 0.03 to 0.3 μg/g of body weight per day in the general European population [13]. The European Commission imposed a limit of 50 μg/g [14] on sunflower oil, but no limit has been set for the concentration of AHCs in seaweeds. Polycyclic aromatic hydrocarbons (PAHs) are another relevant type of pollutants being present in all terrestrial and marine environments; they are considered priority compounds by the United States Environmental Protection Agency [15]. PAHs have received considerable attention due to the fact that some of these compounds have carcinogenic potential [16,17]. Their presence in the aquatic environment results from inadequate disposal of domestic and industrial sewage, river drainage, maritime activities and boat traffic, oil production and transport, fossil fuel burning by vehicles, industrial processes, and atmospheric deposition [18]. Excluding occupationally exposed subjects, exposure to PAHs mainly occurs through ingestion and/or inhalation. Thus, monitoring these compounds in environmental/food samples is an essential step in controlling human exposure [15]. According to the most recent European legislation on the maximum levels of PAHs present in some foods [19], seaweeds are not included, but the maximum levels for fresh marine foods such as bivalves are 5 µg/kg of fresh weight (FW) sample in the case of benzo[a]pyrene (B(a)P) and 30 µg/kg FW for the sum of B(a)P, chrysene (Chry), benzo[a]anthracene (B(a)A), and benzo[b]fluoranthene (B(b)Ft). In the bibliographic research carried out, it was found that studies on the quantification of PAHs, and in particular B(a)P considered the marker of exposure to carcinogenic PAHs, in seaweed are scarce [20,21,22,23].

Since more detailed studies on seaweed composition and bioactivities may lead to new applications targeted to solve the increasingly growing unbalanced dietary habits, the present study aimed to ascertain the nutritional quality of several seaweed species (wild and commercial) through the calculation of specific indices. Besides, the safety of their consumption was also evaluated through the analysis of AHCs and PAHs.

## 2. Materials and Methods

### 2.1. Samples

Seaweed samples were collected between April and November 2016 from the northern shore of Portugal, comprising 10 different species of seaweeds: *Chondrus crispus*, *Gracilaria* spp., *Osmundea pinnatifida* and *Porphyra* spp. (red seaweeds); *Ascophyllum nodosum*, *Fucus spiralis*, *Laminaria ochroleuca*, *Saccorhiza polyschides*, and *Undaria pinnatifida* (brown seaweeds) and one species: *Ulva* spp. (green seaweed). Seaweeds were harvested manually, cutting the fronds, washed with seawater, and transported in plastic bags. In the laboratory, samples were washed with 35 g/L NaCl solution to remove seawater and foreign matter, then rinsed with deionized water and excess water was removed with a paper towel. Afterwards, seaweeds were dried at 42 °C (Excalibur 9 Tray Dehydrator, Model 4926 T, USA) during 6–8 h, stored in polypropylene bag in the dark, and at room temperature until usage. Seven commercially available European dried seaweeds (*C. crispus*, *Porphyra* spp., *A. nodosum*, *Fucus vesiculosus*, *Laminaria* spp., *U. pinnatifida*, *Ulva* spp. were acquired in local stores. Prior to extraction, samples were milled in a mechanical grinder (Moulinex A320) for 5 min to reduce particle size (<5 mm).

### 2.2. Lipid Extraction and Determination of Total Lipids in Seaweed

Lipids were extracted by Soxhlet apparatus (Soxtest, Raypa, Barcelona Spain) according to Crespo et al. [24]. The extract was evaporated, and the lipid content of the sample was calculated by the mass of the dry residue [1]. Each extract was analyzed at least in triplicate.

### 2.3. Fatty Acids Profile Determination

The lipid residue obtained in Section 2.2 was dissolved in 3 mL of n-hexane (Uvasol, Merck, Darmstad, Germany). The color of lipid residue was removed with activated carbon and the fatty acid profile was obtained by base-catalyzed transesterification, according to Bondia-Pons et al. [25].

Gas chromatography analyses were performed on Shimadzu GC-2010 (Kyoto, Japan) equipped with a flame ionization detector (FID) and a Shimadzu AOC-20i auto-injector. Separation of fatty acid methyl ester (FAMEs) was carried out on an Agilent^®^ J&W CP-Sil 88 capillary column (60 m × 0.25 mm I.D., 0.20 µm) from Santa Clara, USA. Operating conditions were as follows: the split-splitless injector was used in split mode with a split ratio of 1:10; the injection volume of the sample was 1.0 µL; the injector and detector temperatures were kept at 250 °C and 260 °C, respectively; the temperature program was as follows: initial temperature 100 °C for 5 min, increased at 1 °C/min to 215 °C and held at this temperature for 20 min (total run time: 140 min). The carrier gas was He (30 mL/min) and the detector gases were H_2_ (40 mL/min) and air (400 mL/min). Data acquisition and processing were performed with Shimadzu software GC solution. The fatty acids content (expressed in %) was obtained from each fatty acid and total fatty acids area ratio. Each extract was analyzed at least in triplicate.

### 2.4. Nutritional Quality Indices

The nutritional quality of the lipids was assessed by considering the following indices: atherogenicity index (AI), thrombogenicity index (TI), hypocholesterolemic/hypercholesterolemic ratio (HH), according to the respective Equations (1)–(3) below. Additionally, the ratio PUFA/saturated fatty acids (SFA) and the ratio n6/n3 were calculated [26].

(1) $$\mathrm{AI}=\frac{C12:0+4\times C14:0+C16:0}{\sum^{\;}\mathrm{MUFA}}$$

(2) TI=C14:0+C16:0+C18:00.5 ×MUFA+0.5 ×n6 PUFA+3×n3 PUFA+n3n6

(3) $$\mathrm{HH}=\frac{\mathrm{cis}\;C18:1+\sum^{\;}\mathrm{PUFA}}{C12:0+C14:0+C16:0}$$

### 2.5. Determination of AHCs

Eight AHCs (Octadecane (C18), Nonadecane (19), Eicosane (C20), Docosan (C22), Tetracosan (C24), Octacosan (C28), Dotriacontano (C32), and Hexatriacontano (C36)) were selected because they had already been described in the literature as present in seaweed in various parts of the world [8]. AHCs were isolated from the lipid extract by activated carbon, submitted to solid-phase extraction (SPE) clean-up [24], and analyzed by GC-FID, with the equipment and column described in Section 2.3. One µL was injected in split mode. The injector and detector temperatures were maintained at 250 °C and 260 °C, respectively. The column temperature was programmed as follows: 1 min at 100 °C, increased to 210 °C at 10 °C min^−1^, held for 6 min, then raised to 220 °C at 10 °C min^−1^, and isothermal for 35 min. AHCs were identified with individual standard solutions. Calibration curves were plotted with seaweed extract fortified after extraction with a mixture of standard solutions. Each extract was analyzed at least in triplicate.

### 2.6. Determination of PAHs

The extractions of PAHs from the seaweeds were carried out by microwave-assisted extraction based on a previously validated methodology [27] Briefly, the optimal extraction conditions were: glass extraction vessels, 1 g of sample, 20 min at 110 °C with 10 mL of acetonitrile and medium agitation. The PAHs quantification was carried out by HPLC-FLD/PAD according to Ramalhosa et al. [28]. The PAHs present in the standard mixture (Supelco, Bellefonte, PA, USA) were: naphthalene (Naph), acenaphthylene (Acen), acenaphthene (Ace), fluorene (Flu), phenanthrene (Phe), anthracene (Ant), fluoranthene (Fln), pyrene (Pyr), benz(a)anthracene (B(a)A), chrysene (Chry), benzo(b)fluoranthene (B(b)Ft), benzo(k)fluoranthene (B(k)Ft), benzo(a)pyrene (B(a)P), dibenz(a,h)anthracene (DB(a,h)A), benzo(g,h,i)perylene (B(g,h,i)P), indeno(1,2,3-cd)pyrene (InP) and dibenzo(a,l)pyrene (D(a,l)P). Each extract was analyzed at least in triplicate.

### 2.7. Statistical Analysis

Statistical analyses were performed with IBMS SPSS for Windows, version 26 (IBM Corp., Armonk, NY, USA). The data normality was assessed by Kolmogorov–Smirnov, and Shapiro–Wilk tests, and by visual inspection of histograms. Elemental concentrations were expressed as mean ± standard deviation. For each seaweed species, comparisons between groups were made using the Mann-Whitney test, at a level of significance of *p* < 0.05.

## 3. Results and Discussion

### 3.1. Total Lipids in Seaweeds

The amount of total lipids (% in dry mass) in the different species of seaweeds (Table 1) varied between 0.7 ± 0.1% (*C. crispus*) and 2.5 ± 0.6% (*S. polyschides*). There were no significant differences between red, brown, and green seaweeds. Regarding commercial seaweeds, total lipid content varied between 1.4 ± 0.2 (*C. crispus*) and 3.8 ± 0.1% (*Ulva* spp.), *Ulva* spp. presented higher (*p* < 0.05) values than wild seaweeds.

Comparing the results obtained in the present work with those reported in the literature (Table 1), differences were observed for some seaweed species, particularly for *F. spiralis*, *Ulva* spp., *L. ochroleuca*, and *Gracilaria* spp. Different analytical methodologies can affect the lipid composition. According to Nelson et al. [37], the lipid content in seaweeds is dependent on the season, also varying according to geographic location, climate and environmental conditions, such as temperature, salinity, and nutrients. As presented in Table 1, *A. nodosum* is the seaweed specie with generally the highest lipid content (1.5 to 3.2% DW) while *C. crispus* presented the lowest lipid content (0.70 to 2.0% DW).

### 3.2. Lipidic Profile of Seaweed

Seaweeds present distinct fatty acid profiles, depending on their habitat, harvest season, and genetics [38]. Among the species analyzed, PUFA ranged between 0 to 35% of the total fatty acids (see Tables S1 and S2 in Supplementary Material), with *Ulva* spp. collected in Autumn, presenting the highest amount (35%). Monounsaturated fatty acids (MUFA) varied between 19 and 67%, and SFA were predominant in *C. crispus* (45–78%) and *Gracilaria* spp. (36–79%). It must be noted that such significant content variations for the same species were related to the harvest season: the lowest SFA values were consistently correlated with autumn when compared with values of catches during spring or summer. Other authors [39] also reported that the PUFA content and degree of unsaturation of seaweeds harvested in cold regions (Canada) were higher than those harvested in southern China’s tropical waters and Indo-Pacific region. The above statement is justified by the known correlation between environmental temperature and unsaturated fats, aiming to maintain membrane fluidity and thus normal functioning [40]. In our study, for a specific seaweed species, the lowest SFA recorded always corresponded to higher amounts of MUFA or PUFA (Tables S1 and S2 in Supplementary Material). It is important to note that these profiles vary in the same species, with the geographical location and time of the year in which the harvest was carried out and the storage time [41]. The sum of SFA, MUFA, and PUFA showed higher values for *Gracilaria* spp. in spring/summer, A. nodosum in both seasons, and *Ulva* spp. in autumn/winter, respectively. The total levels of omega-3 and omega-6 families were higher for *O. pinnatifida* (30.52%) and *S. polyschides* (18.30%), respectively, both in the season corresponding to lower ambient temperatures (autumn/winter).

It was found that the lipid quality indices vary between species and, within the same species, with the season. Among the several species studied, *A. nodosum* presented the best indices of lipid quality. Because of the previous statement, the analysis of each fatty acid may vary considerably for as for the same species collected in the same region. Values can be quite distinct, depending on the season: *Porphyra* spp. was collected during summer and autumn seasons, and its content in EPA ranged between 19 and 27%. When dealing with commercial species, this uncertainty can grow even more due to a lack of knowledge on the harvesting season and remaining conditions; indeed, the commercial *Porphyra* spp. analyzed contained only 1.9% of EPA. For the above reasons, it is preferable to evaluate the nutritional/functional/bioactivity potential of seaweeds relying on indices that consider several FA and the ratios among them. Furthermore, seaweeds are a source of healthy lipids and may accumulate high contents of SFA (e.g., palmitic acid, C16:0), which may be undesirable for human consumption [7]. Therefore, it is preferable to evaluate the nutritional impact of lipid seaweeds using lipid quality indices.

### 3.3. Nutritional Indices of Seaweed

From the selected indices, the AI indicates the relationship between the sum of the main SFA—considered as pro-atherogenic—and the main classes of unsaturated fatty acids (UFA)—considered as anti-atherogenic (inhibiting the aggregation of plaque, thereby preventing the appearance of micro and macro coronary diseases). The TI index denotes the tendency to form clots in blood vessels as a ratio between the pro-thrombogenic (saturated) and the anti-thrombogenic fatty acids. PUFA/SFA is the ratio most employed to evaluate the influence of diet on cardiovascular health, and HH ratio was developed as a more accurate version to measure the effect of fatty acid composition on cholesterol [26]. In addition, the n6/n3 ratio, a balance between anti-inflammatory and pro-inflammatory metabolic precursors (eicosanoids), is currently recommended by the World Health Organization to be lower than 10 in the diet (order to prevent inflammatory and cardiovascular disorders) [39].

Although some AI values measured in our study (see Tables S1 and S2 in Supplementary Material) were higher than those in the literature for some of the species (e.g., we recorded AI between 0.61–1.02 and 0.59 for wild and commercial *U. pinnatifida*, respectively, where Dellatorre et al. [42] report values ranging 0.17–0.35), others were lower than literature (we recorded 0.49–0.80 for *L. ochroleuca*, as compared with 1.18–1.57 from Otero et al. [7]. Moreover, compared with some usual dietary list components (e.g., lamb, with AI 0.99–1.32), seaweed species present a more healthy index [43]. Reported AI values for some foods ranged between 0.084 and 0.55 for crops, 0.21 and 1.41 for fish, and 0.165 and 1.32 for meat [26]. Values between 1.42 and 5.13 were described for dairy products [26].

For the studied seaweeds, the TI value ranges from 0.04 to 1.95, except for *Gracilaria* spp., which had a TI of 7.61. *Gracilaria* species are reported to present higher TI values, such as *G. salicornia*, which had a TI value of 5.75 [44]. Again, the TI values recorded in our *L. ochroleuca* were substantially lower than those reported in the literature: 0.14–0.23 vs. 1.06–1.89 [7]. TI values for crops, fish, meat, and dairy products are reported as 0.139–0.56, 0.14–0.87, 0.288–1.694, and 0.39–5.04, respectively [26].

Unlike the previous indices, where the lower the value the better, for HH ratio, the highest value indicates a lower cholesterolemic impact. HH values in our study ranged 0.88–4.21, except for *Gracilaria* spp., which exhibited values between 0.22–9.26. HH was used as one of the indices to assess the nutritional and health-promoting properties of seaweed from the Azores, and values concerning *Ulva* species ranged from 1.26 to 2.09 [2], comparable with our results 0.97–1.54. Values in the range of 1.54 to 4.83 were reported for fish, whereas values between 1.27–2.786, and 0.32–1.29 were described for meat and dairy products, respectively [26].

It can be observed that n6/n3 is below 1 for most of the species evaluated, except *A. nodosum*, which presented a higher ratio, although below 2 (Tables S1 and S2 in Supplementary Material).

PUFA/SFA ratio is generally used to assess the nutritional value of foods such as seaweeds (0.42–2.12, aside from *Gracilaria changii*), meat (0.11–2.042), fish (0.50–1.62), shellfish (0.20–2.10), and dietary products (0.02–0.175) [26]. Our results pointed out values between 0.11–1.02, (except for *Gracilaria* spp.).

From the abovementioned results of nutritional indices, it is possible to conclude that an appropriate choice of seaweed species may become a promising strategy to enhance food quality in terms of its bioactive properties, for example, to prevent inflammatory, cardiovascular diseases and nervous system disorders. These indices also indicate that these seaweeds provide a vegetarian and vegan-friendly source of healthy fats comparable to some fish species.

### 3.4. AHCs in Seaweeds

**Figure 1:**
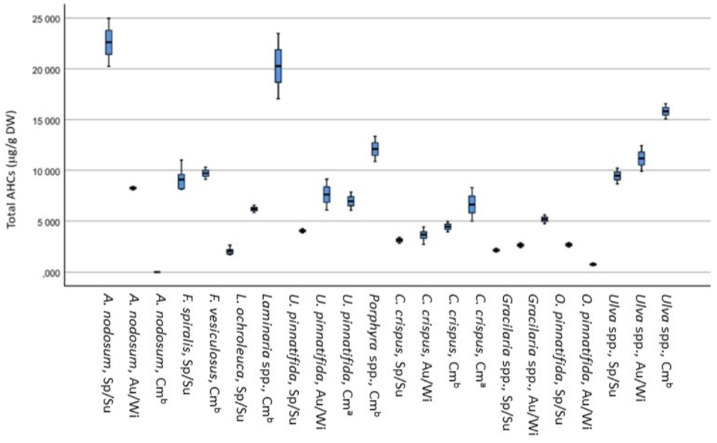
Box plots showing the Total AHCs content (ug/g DW) in different seaweeds species. Sp: Spring; Su: Summer; Au; Autumn; Wi: Winter; Cm ^a^: Commercial wild origin; Cm ^b^: Commercial aquaculture origin.

The 10 seaweeds harvested at different times of the year were analyzed for AHCs content. The results obtained for the total AHCs content are presented in Figure 1.

The total AHCs content varied between 11 and 32 µg/g DW in *A. nodosum*, 2.8 and 13 μg/g DW in *C. crispus*, 11 and 13 μg/g DW in *Fucus* spp., 3.6 and 7.0 μg/g DW in *Gracilaria* spp., 2.4 and 19 μg/g DW in *Laminaria* spp., 1.0 and 19 μg/g DW in *O. pinnatifida*, 4.1 and 17 μg/g DW in *Porphyra* spp., 13 and 21 μg/g DW in *Ulva* spp. and 5.4 and 28 μg/g DW in *U. pinnatifida*.

Between each species, some differences were observed according to the sampling station. For example, *Gracilaria* spp. has a higher total AHCs in autumn (7.0 µg/g DW) than spring (3.6 µg/g DW). *Porphyra* spp. presents a value of 9.6 µg/g for the summer harvest, increasing AHC’s concentration in the autumn harvest, presenting a concentration of 17 µg/g. The *U. pinnatifida* in the summer contained a concentration of 28 µg/g, and in the winter, it only presented 5.4 µg/g DW. The results obtained also show that AHCs with a higher number of carbons (C32 and C36) were less frequent in seaweed and are present at lower levels (see Supplementary Material, Table S3).

Comparing the results obtained with those in the literature for total AHCs, *A. nodosum* presents higher values (11–32 µg/g DW) than the values reported by Clark and Blumer [45], which was 6.96 µg/g DW. Crespo et al. [24] analyzed *U. pinnatifida* samples collected at different dates and areas of the Galician coast. The n-alkanes C18, C20, C22, C24, and C28 were found in all samples, with values lower than 7.9 μg/g DW and the total hydrocarbon content was within the range of 13.6–21.7 μg/g DW. These values were like the ones found in this study for the same seaweed (5.4 to 28 μg/g DW). C18 was found to be the most abundant [24]. This hydrocarbon is also the most common in the seaweeds reported in this study (not detected (n.d.) to 12 μg/g DW), followed by C19 (0.16 to 5.7 μg/g DW). No study was found on the Portuguese coast for comparative analysis.

Considering the limits imposed by the European Commission for AHCs in sunflower oil as a reference (50 µg/g DW) [14], the seaweeds harvested along the central and northern Portuguese coast are below this limit, with the highest value obtained (12 µg/g DW) for the C18 AHCs in *A. nodosum*.

Thus, it is possible to conclude that the seaweeds assessed in this study are safe for human consumption and, due to their nutritional and functional features, they should be part of a wholesome diet.

### 3.5. PAHs in Seaweeds

Table 2 shows the PAHs contents quantified in the characterized wild seaweed species. As can be observed, it was possible to quantify some of the compounds, namely Fln and Pyr in *F. spiralis* harvested during spring and Pyr in autumn. For *Porphyra* spp., Pyr and B(a)A were detected in the seaweeds harvested in spring and Fln in winter. For *Ulva* spp., Phe was identified in the harvest of Spring and Ace in the harvest of Summer. For the other samples and other PAHs (18 in total were analyzed), the results were lower than the LOD of the various compounds and therefore not included in Table 2 and Table 3. Concerning the four PAHs considered in the legislation (B(a)P, Chry, B(a)A, and B(b)Ft), only B(a)A was found in *Porphyra* spp. collected in summer with 0.11 µg/kg FW, with a value much lower than the recommended limit of 30 µg/kg FW for the sum of the 4 PAHs indicated. Comparing the obtained data with those previously published for wild seaweeds, the total PAHs found in the different harvests were much lower than those of Kirso et al. [20] and very similar in the various seaweeds groups, probably due to the low pollution of the Portuguese coast.

Kirso et al. [20] analyzed PAHs in the waters, sediments, and seaweeds of the Baltic Sea in polluted areas. These authors reported that the most abundant PAHs were found in *F. vesiculosus* with Pyr at 341 µg/kg DW, followed by D(a,l)P with 30 µg/kg DW, B(g,h,i)P with 12.5 µg/kg DW to a total PAHs content of 468.9 µg/kg DW. B(a)P had a content of 0.7 µg/kg DW. According to these authors, the distribution of PAHs in water and seaweeds was very similar and much lower than the levels found in sediments, which led them to conclude that seaweeds were exposed to PAHs through the water column and not through the rhizoid in contact with sediments [20]. They also compared the bioaccumulation capacity of B(a)P by seaweed species and concluded that brown seaweeds could bioaccumulate large amounts of PAHs compared to green and red seaweeds, since these two groups have enzymes capable of metabolizing PAHs at the same time, contrary to brown seaweeds. In another study carried out in the Venice lagoon [21], the PAHs identified in the samples were mainly those consisting of 3–4 aromatic rings. Phe and Ant were found in all samples corresponding to 14% of the total PAHs, Fln found in 97% of the samples being on average 22% of the total PAHs, Pyr detected in 97% of the cases and corresponding to 28% of the total PAHs. PAHs with two aromatic rings, such as Acen, Ace, and Flu, were rarely more than 10% of the total PAHs and were detected in about 40% of the samples. The differences found in the type of PAH quantified were dependent on the species of seaweed analyzed and the sampling site. In most of the species reported in the literature [21], Fln and Pyr are the PAHs present in greater quantity, being in accordance with the PAHs identified in the seaweeds harvested in Portugal (Table 2).

The commercial seaweeds acquired within the scope of this study were also analyzed, and the levels of PAHs obtained are shown in Table 3. It is possible to verify that *U. pinnatifida* and *A. nodosum* presented the highest content of PAHs analyzed in this work: 1.0 ± 0.1 and 0.93 ± 0.18 µg/kg FW, respectively. Regarding the PAHs indicated in the legislation, only B(b)Ft was quantified in the commercial *Ulva* spp., but the value found (0.14 µg/kg FW) was much lower than the maximum limit recommended by the legislation (30 µg/kg FW) for other foodstuff.

Concerning the PAHs identified, Phe and Fln were the most detected (Table 3). Recently it was reported in Portugal that commercially available *Ulva* spp. contained concentrations of PAHs up to 51.8 µg/kg DW in the dried samples, while the fresh samples contained 0.27 µg/kg DW. However, considering measured values and bioaccessibility of PAHs, the authors concluded them to be safe for consumers [22]. Samples of different seaweed species (browns and reds) used for human consumption from Spain showed PAHs concentrations below 200 mg/kg DW [23]. B(a)P was not found in the samples by these authors. It was concluded that seaweed consumption concerning PAH content does not pose a human health concern [23].

PAHs can be found in seaweeds; however, the few studies with species proposed for consumption showed concentrations that do not pose a human health concern. However, possible influencing factors on the presence of PAHs include the cultivation environment, especially the proximity to industrial or anthropogenic activities, or if occurring after significant events such as an oil spill, the influence of processing (for example, drying) on the seaweed, and the accumulation capacity of PAHs in the seaweed [46].

## 4. Conclusions

It is possible to conclude that the seaweeds studied are safe for human consumption and, due to their nutritional and functional properties, they should be included in a healthy diet. The seaweeds analyzed in this study present a total lipid content between 0.7 to 3.8% DW with *Ulva* spp. being the seaweed with the highest lipid content (1.2 to 3.8% DW) and *C. crispus* with the lowest (0.70 to 2.0% DW). Among the species analyzed, PUFA ranged between 0–35%, with *Ulva* spp. presenting the highest amount, MUFA varied between 19 and 67%, and SFA were predominant in *C. crispus* (45–78%) and *Gracilaria* spp. (36–79%). Concerning the nutritional indices calculated for the seaweeds collected and acquired in Portugal, AI was between 0.4–3.2, which can be considered a healthy index. For TI, the value ranged from 0.04 to 1.95, except for *Gracilaria* spp., which had a TI of 7.6. HH values in this study ranged between 0.88–4.21, except for *Gracilaria* spp., which exhibited values between 0.22–9.26. The n6/n3 ratio is below 1 for most of the species evaluated, except for *A. nodosum*, which presented a higher ratio, although below 2. Considering the PUFA/SFA ratio used to assess the nutritional value, the seaweeds analyzed in this study pointed out values between 0.11–1.02 (except for *Gracilaria* spp.). These results indicate that *Ulva* spp. that presents the highest content of PUFA and MUFA and *A. nodosum* with the best indices of lipid quality are the most interesting seaweeds for human nutrition considering the bioactive lipids. However, it is necessary to consider that the scope of this work is only the lipophilic portion of seaweeds, but the levels of other organic (dioxins, polychlorinated biphenyls, pharmaceuticals, etc.) and inorganic pollutants (heavy metals), as well as other essential elements that exist at high levels in seaweeds (e.g., iodine, it can be harmful to health if excessively consumed) must be assessed to perform a comprehensive characterization of food safety.

Considering the possible lipophilic contaminants assessed in this study, the AHCs detected in the analyzed seaweeds are at very low levels, which are not considered harmful for human health. PAHs can be found in seaweed; however, the values found in seaweed do not pose a human health concern. However, the creation of appropriate legislation must be reinforced to establish safe limits of PAH exposure for these kinds of products. In addition, further research is needed to assess the risk of food safety hazards in seaweed.

## Figures and Tables

**Table 1:** Quantity of total lipids (mean ± SD % DW) in different seaweeds species and comparison with values reported in the literature.

Seaweed Collected	Species	Total Lipids (% DW)		
		This Work	Literature	Season, Year, Reference
Su	*A. nodosum*	2.4 ± 0.1 b	3 ± 1	Sp/Su 2012, [29]
Au		1.5 ± 0.2 c	2 ± 1	Au/Wi 2011, [29]
Cm ^a^		3.2 ± 0.4 a		
Su	*C. crispus*	1.2 ± 0.5 b	0.9 ± 0.2	Sp/Su 2012, [29]
Au		0.7 ± 0.1 c	0.9 ± 0.2	Au/Wi 2011, [29]
Cm ^a^		2.0 ± 0.1 a		
Cm ^b^		1.4 ± 0.2 b		
Su	*F. spiralis*	2.0 ± 0.9 a		
Au		2.1 ± 0.1 a	5.23 ± 0.03; 1.87	Au/Wi 2013, [2]; Au/Wi 2007, [30]
Cm ^a^	*F. vesiculosus*	2.9 ± 0.1		
Sp	*Gracilaria* spp.	2.0 ± 0.1 a	0.60 ± 0.01	Sp/Su 2012, [31]
Au		0.80 ± 0.07 b	1.12	Au/Wi 2012, [32]
Sp	*L. ochroleuca*	2.2 ± 0.2	0.92 ± 0.01	Sp/Su 2001, [33]
Cm ^a^	*Laminaria* spp.	1.8 ± 0.4		
Su	*O. pinnatifida*	1.9 ± 0.1 a	0.9 ± 0.1	Sp/Su 2012, [31]
Wi		1.7 ± 0.4 a	7.53± 0.07; 2.58	Au/Wi 2013, [2]; Au/Wi 2007, [30]
Su	*Porphyra* spp.	2.3 ± 0.4 a	1.03 ± 0.04	Sp/Su 2001, [33]
Au		1.3 ± 0.7 b	3.34	Au/Wi 2007, [30]
Cm ^b^		1.8 ± 0.3 ab		
Su	*S. polyschides*	2.5 ± 0.6 a	1.1 ± 0.1	Sp/Su 2012, [31]
Au		1.0 ± 0.1 b		
Su	*Ulva* spp.	2.0 ± 0.2 b	2.62 ± 0.04	*U. armoricana,* Sp/Su 2012, [34]
Au		1.2 ± 0.1 c	3.14 ± 0.0	Au/Wi 2010, [35]
Cm ^b^		3.8 ± 0.1 a		
Su	*U. pinnatifida*	2.1 ± 0.4 ab	1.05 ± 0.01	Sp/Su 2012, [31]
Wi		0.83 ± 0.06 b	5.1	Au/Wi 2011, [36]
Cm ^a^		2.2 ± 0.4 a		

Sp: Spring; Su: Summer; Au; Autumn; Wi: Winter; Cm ^a^: Commercial wild origin; Cm ^b^: Commercial aquaculture origin. For each seaweed species, distributions with different letters (a–c) are statistically different at *p* < 0.05.

**Table 2:** PAHs levels detected in the seaweeds µg/kg DW (µg/kg FW).

Seaweed	*F. spiralis*		*Porphyra* spp.		*Ulva* spp.	
Harvest Date	Summer	Autumn	Summer	Winter	Spring	Summer
PAH	Mean ± SD µg/kg DW (Mean ± SD µg/kg FW)					
Ace	n.d.	n.d.	n.d.	n.d.	n.d.	1.9 ± 0.1 (0.17 ± 0.01)
Phe	n.d.	n.d.	n.d.	n.d.	0.52 ± 0.03 (0.0311 ± 0.0002)	n.d.
Fln	0.71 ± 0.02 (0.19 ± 0.01)	n.d.	n.d.	0.47 ± 0.02 (0.027 ± 0.001)	n.d.	n.d.
Pyr	0.51 ± 0.02 (0.14 ± 0.01)	0.33 ± 0.01 (0.076 ± 0.003)	2.9 ± 0.1 (0.54 ± 0.01)	n.d.	n.d.	n.d.
B(a)A	n.d.	n.d.	0.61 ± 0.05 (0.11 ± 0.01)	n.d.	n.d.	n.d.
∑ PAHs	1.2 ± 0.1 (0.33 ± 0.01)	0.33 ± 0.01 (0.076 ± 0.003)	3.6 ± 0.1 (0.65 ± 0.02)	0.47 ± 0.02 (0.027 ± 0.001)	0.52 ± 0.03 (0.031 ± 0.002)	1.9 ± 0.1 (0.17 ± 0.01)

n.d.: not detected.

**Table 3:** PAHs levels detected in commercial seaweeds µg/kg DW (µg/kg FW).

Seaweed	*A. nodosum*	*C. crispus*	*F. vesiculosus*	*Porphyra* spp.	*Ulva* spp.	*U. pinnatifida*
Cultivation	Wild	Wild	Wild	Aquaculture	Aquaculture	Wild
PAH	Mean ± SD µg/kg DW (Mean ± SD µg/kg FW)					
Naph	1.6 ± 0.5 (0.50 ± 0.14)	n.d.	n.d.	n.d.	n.d.	n.d.
Flu	n.d.	n.d.	n.d.	n.d.	n.d.	0.29 ± 0.02 (0.044 ± 0.003)
Phe	1.4 ± 0.1 (0.43 ± 0.03)	0.61 ± 0.01 (0.11 ± 0.01)	1.1 ± 0.1 (0.19 ± 0.01)	n.d.	n.d.	3.3 ± 0.1 (0.50 ± 0.06)
Fln	n.d.	n.d.	n.d.	0.43 ± 0.10 (0.065 ± 0.007)	n.d.	1.5 ± 0.1 (0.23 ± 0.03)
Pyr	n.d.	n.d.	n.d.	n.d.	n.d.	1.6 ± 0.2 (0.25 ± 0.03)
B(b)Ft+B(j)Ft	n.d.	n.d.	n.d.	n.d.	1.4 ± 0.1 (0.14 ± 0.01)	n.d.
∑PAHs	3.1 ± 0.6 (0.93 ± 0.18)	0.61 ± 0.01 (0.11 ± 0.01)	1.1 ± 0.1 (0.19 ± 0.01)	0.43 ± 0.10 (0.065 ± 0.007)	1.4 ± 0.1 (0.14 ± 0.01)	6.8 ± 0.1 (1.0 ± 0.1)

n.d.: not detected.

## Data Availability

Data is contained within the article or supplementary material.

## Supplementary Materials

The following are available online at https://www.mdpi.com/article/10.3390/foods10061366/s1, Table S1: Experimental values for the lipid profile and quality indices in the harvested seaweeds, Table S2: Experimental values for the lipid profile and quality indices of the commercial seaweeds; Table S3: Concentrations of aliphatic hydrocarbons (µg/g dry mass) and respective deviations found in seaweeds under study.
