# Supplementary material for: Bioactive Lipids of Seaweeds from the Portuguese North Coast: Health Benefits versus Potential Contamination
Source: Foods. 2021 Jun 12;10(6):1366. doi: 10.3390/foods10061366 (PMC8231286; doi:10.3390/foods10061366)
Supplement: Supplementary file 1 [file foods-10-01366-s001.zip › foods-1225628-supplementary.pdf]

Table S1. Experimental values for the lipid profile and quality indices in the harvested seaweeds.

**Seaweeds**	***A. nodosum***						***C. crispus***						***F. spiralis***					
**Harvest**	Summer		Autumn		Winter		Spring		Summer		Autumn		Spring		Summer		Autumn	
**Fatty acids**	%	SD	%	SD	%	SD	%	SD	%	SD	%	SD	%	SD	%	SD	%	SD
**SFA**																		
C12:0					9.9E-04	1.0E-05												
C13:0																		
C14:0	5.6E-02	4.9E-03	5.6E-02	1.4E-03	7.3E-02	5.4E-03	1.5E-02	1.2E-03					9.8E-02	9.2E-03	9.8E-02	8.7E-04	9.8E-02	1.4E-03
C15:0			1.6E-03	2.2E-04	2.1E-03	3.6E-05							2.5E-03	1.8E-04	2.5E-03	1.1E-04	2.9E-03	3.5E-05
C16:0	1.0E-01	1.6E-03	7.6E-02	1.4E-03	1.2E-01	8.2E-03	1.9E-01	1.7E-02	2.5E-01	5.9E-02	2.6E-01	1.5E-02	1.4E-01	2.2E-03	1.3E-01	6.6E-05	1.5E-01	1.0E-03
C17:0			1.8E-03	1.2E-04	2.1E-03	4.4E-05							5.5E-03	4.8E-04	5.2E-03	1.7E-05	8.1E-03	5.6E-04
C18:0	1.9E-02	3.1E-03	1.2E-02	1.7E-04	2.5E-02	5.6E-04	4.7E-01	3.3E-02	5.2E-01	1.1E-01	1.9E-01	4.8E-02	2.5E-02	8.5E-05	2.4E-02	1.9E-04	2.0E-02	7.2E-04
C20:0			5.2E-03	4.5E-04	4.3E-03	2.1E-04							4.0E-03	2.0E-04	2.6E-03	8.3E-05	4.3E-03	1.1E-04
C21:0																		
C22:0	5.3E-03	1.6E-03	9.1E-03	2.1E-03	6.4E-03	3.5E-04							7.0E-03	3.2E-04	6.7E-03	6.6E-04	8.1E-03	1.1E-04
C23:0			5.8E-03	3.0E-04	4.9E-03	1.1E-04												
C24:0			1.9E-03	1.7E-04	3.5E-03	1.9E-04							2.7E-03	7.0E-05	1.7E-03	1.2E-04	2.2E-03	4.4E-06
**MUFA**																		
C14:1 n-5			2.8E-03	5.1E-04	3.2E-03	2.6E-04											1.4E-03	1.8E-05
C15:1 n-5																		
C16:1 n-7	1.3E-02	3.6E-03	1.5E-02	4.7E-04	2.1E-02	1.5E-03	1.1E-02	1.7E-04					1.2E-02	6.2E-04	1.1E-02	1.9E-05	1.4E-02	2.6E-05
C17:1 n-7					1.1E-03	4.9E-05											5.8E-04	3.2E-05
C18:1 n-9 t																		
C18:1 n-9 c	5.3E-01	4.0E-02	5.3E-01	5.5E-03	4.0E-01	3.0E-03	1.0E-01	5.0E-03	1.2E-01	9.9E-03	1.6E-01	2.6E-02	4.1E-01	1.5E-02	4.2E-01	1.1E-02	3.6E-01	3.8E-03
C20:1n-9					1.7E-02	8.7E-04												
C22:1 n-9	1.3E-01	1.7E-02	1.5E-01	2.7E-04	1.1E-01	5.8E-03	1.3E-01	5.0E-03	1.4E-01	2.9E-02	2.6E-01	7.6E-03	1.5E-01	5.3E-03	1.5E-01	5.3E-03	1.5E-01	1.8E-03
C24:1 n-9																		
**PUFA**																		
C18:2 n-6 t																		
C18:2 n-6 c	7.0E-02	9.3E-03	7.1E-02	4.2E-04	9.9E-02	8.0E-03							5.7E-02	4.1E-04	5.7E-02	1.8E-05	6.3E-02	2.2E-04
C18:3 n-3	1.7E-02	1.6E-03	1.5E-02	9.9E-04	3.8E-02	4.1E-04							3.1E-02	5.0E-04	3.2E-02	3.8E-04	4.9E-02	1.7E-03
C18:3 n-6			2.4E-03	3.7E-04	2.8E-03	7.0E-05							7.4E-03	3.7E-04	7.1E-03	8.9E-05	6.8E-03	5.4E-04
C20:2 n-6	9.2E-03	1.4E-03	1.0E-02	1.4E-03	1.3E-02	1.7E-04									3.1E-03	7.8E-05	4.0E-03	4.8E-05
C20:3 n-3			2.7E-03	1.9E-04											2.8E-03	4.2E-04	1.3E-03	9.5E-05
C20:3 n-6	6.2E-03	1.8E-04	1.3E-03	7.9E-05	2.3E-03	4.9E-05							2.4E-03	1.8E-04	2.1E-03	2.3E-04	2.2E-03	7.8E-05
C20:4 n-6																		
C20:5 n-3	3.5E-02	5.3E-03	3.5E-02	1.1E-04	3.4E-02	2.8E-04	7.6E-02	4.2E-03	1.1E-01	3.9E-02	1.3E-01	2.9E-02	4.5E-02	3.0E-03	4.5E-02	3.6E-03	6.0E-02	1.6E-03
C22:2 n-6																		
C22:6 n-3					7.3E-03	5.1E-04												

**ΣSFA**	1.8E-01		1.7E-01		2.4E-01		6.8E-01		7.7E-01		4.6E-01		2.8E-01		2.8E-01		2.9E-01	
**ΣMUFA**	6.8E-01		6.9E-01		5.6E-01		2.4E-01		2.6E-01		4.2E-01		5.8E-01		5.8E-01		5.2E-01	
**ΣPUFA**	1.4E-01		1.4E-01		2.0E-01		7.6E-02		1.1E-01		1.3E-01		1.4E-01		1.5E-01		1.9E-01	
**Σω3**	5.2E-02		5.3E-02		8.0E-02		7.6E-02		1.1E-01		1.3E-01		7.5E-02		8.0E-02		1.1E-01	
**Σω6**	7.6E-02		7.5E-02		1.2E-01								6.7E-02		6.6E-02		7.2E-02	
**AI**	4.0E-01		3.7E-01		5.5E-01		8.0E-01		6.9E-01		4.8E-01		7.4E-01		7.3E-01		7.6E-01	
**TI**	3.3E-01		2.9E-01		1.7E-01						1.1E+00		1.5E-01		4.5E-01		4.1E-01	
**HH**	4.1E+00		4.9E+00		3.0E+00		8.4E-01		9.2E-01				2.3E+00		2.4E+00		2.2E+00	

Table S1. Experimental values for the lipid profile and quality indices in the harvested seaweeds (Cont.).

**Seaweeds**	***Gracilaria* spp.**				***L. ochroleuca***				***O. pinnatifida***							
**Harvest**	Spring		Autumn		Spring		Summer		Spring		Summer		Autumn		Winter	
**Fatty acids**	%	SD	%	SD	%	SD	%	SD	%	SD	%	SD	%	SD	%	SD
**SFA**																
C14:0	4.8E-02	5.5E-03	4.8E-02	3.7E-04	4.4E-02	1.7E-03	3.9E-02	8.0E-04	3.3E-02	1.7E-03	3.1E-02	1.5E-03	4.5E-02	1.5E-03	3.1E-02	5.7E-03
C15:0							2.6E-03	1.6E-04	3.5E-03	1.0E-05			5.4E-03	2.4E-04		
C16:0	4.7E-01	9.2E-03	2.8E-01	1.2E-02	2.9E-01	1.9E-02	1.9E-01	2.9E-03	2.6E-01	2.8E-02	3.1E-01	2.2E-02	2.3E-01	1.1E-02	3.0E-01	1.4E-02
C17:0							2.4E-03	8.6E-07	2.2E-03	5.8E-05			2.1E-03	1.5E-04		
C18:0	2.8E-01	1.8E-02	3.4E-02	9.8E-03	5.9E-02	4.8E-03	5.2E-02	4.4E-03	5.7E-02	4.1E-03	8.2E-02	9.0E-03	6.2E-02	5.4E-03	3.4E-02	3.3E-03
C20:0					5.4E-03	2.7E-04	3.7E-03	2.7E-04	2.4E-03	2.0E-04			1.9E-03	9.9E-05	7.0E-03	4.0E-03
C21:0																
C22:0					5.6E-03	5.6E-04	3.4E-03	9.0E-05	2.7E-03	1.3E-04					5.0E-03	4.0E-04
C23:0																
C24:0							2.3E-03	1.8E-05								
**MUFA**																
C14:1 n-5																
C15:1 n-5			4.5E-01	3.4E-02												
C16:1 n-7					4.3E-02	4.2E-03	4.2E-02	3.3E-03	5.1E-02	3.5E-03	3.6E-02	6.2E-03	6.4E-02	2.9E-03	1.5E-02	5.8E-03
C17:1 n-7							1.3E-03	4.3E-05	2.2E-03	1.0E-04						
C18:1 n-9 t							2.8E-03	2.3E-05	6.3E-03	1.1E-03						
C18:1 n-9 c	1.2E-01	1.0E-02	7.3E-02	1.1E-02	2.4E-01	3.4E-03	2.9E-01	6.1E-04	2.8E-01	2.0E-02	2.8E-01	2.5E-02	2.6E-01	5.4E-03	1.5E-01	4.7E-03
C20:1n-9	2.1E-02	2.6E-03			3.8E-03	1.4E-04	2.3E-02	2.9E-04	3.4E-02	2.6E-03			5.5E-02	4.5E-03		
C22:1 n-9	5.3E-02	4.2E-03	1.2E-01	1.9E-03	1.7E-01	1.1E-02	4.7E-02	2.3E-03	1.7E-02	7.5E-05	9.5E-02	7.3E-03	1.3E-02	1.1E-03	1.4E-01	6.9E-03
C24:1 n-9							2.8E-03	1.6E-04	4.1E-03	2.0E-05			5.4E-03	3.2E-04		
**PUFA**																
C18:2 n-6 t																
C18:2 n-6 c	1.5E-02	1.7E-04			5.7E-02	2.2E-03	1.2E-01	3.3E-03	9.6E-02	1.0E-02	1.1E-02	1.1E-03	9.9E-02	2.0E-03	1.7E-02	3.1E-03
C18:3 n-3					1.7E-02	3.5E-04	8.9E-02	7.5E-03	4.6E-02	1.3E-02			3.4E-02	1.9E-03		
C18:3 n-6					5.4E-03	8.1E-04	3.4E-03	4.4E-05	2.0E-03	2.9E-05			2.6E-03	2.2E-04		
C20:2 n-6							2.9E-03	1.4E-04	2.8E-03	8.7E-05						
C20:3 n-3																
C20:3 n-6							1.3E-03	4.9E-05								
C20:4 n-6							1.9E-03	4.3E-05	3.2E-03	7.1E-06			5.6E-03	1.4E-04		

C20:5 n-3					5.9E-02	2.3E-03	4.0E-02	1.9E-03	4.8E-02	6.6E-03	1.6E-01	7.0E-03	5.1E-02	2.5E-03	3.1E-01	2.7E-03
C22:2 n-6																
C22:6 n-3							3.8E-02	3.4E-03	4.6E-02	4.0E-03			5.8E-02	2.4E-03		
**ΣSFA**	7.9E-01		3.6E-01		4.1E-01		3.0E-01		3.6E-01		4.2E-01		3.5E-01		3.7E-01	
**ΣMUFA**	1.9E-01		6.4E-01		4.5E-01		4.1E-01		4.0E-01		4.1E-01		4.0E-01		3.0E-01	
**ΣPUFA**	1.5E-02		0.0E+00		1.4E-01		3.0E-01		2.5E-01		1.7E-01		2.5E-01		3.2E-01	
**Σω3**			0.0E+00		7.6E-02		1.7E-01		1.4E-01		1.6E-01		1.4E-01		3.1E-01	
**Σω6**	1.5E-02		0.0E+00		6.2E-02		1.3E-01		1.0E-01		1.1E-02		1.1E-01		1.7E-02	
**AI**	3.2E+00		7.4E-01		7.9E-01		4.9E-01		6.1E-01		7.5E-01		6.3E-01		6.8E-01	
**TI**	7.6E+00				7.9E-01		1.3E-01		1.7E-01		5.2E-01		1.7E-01		2.9E-01	
**HH**	2.6E-01		2.1E-01		1.1E+00		2.5E+00		1.8E+00		1.3E+00		1.8E+00		1.4E+00	

Table S1. Experimental values for the lipid profile and quality indices in the harvested seaweeds (Cont.).

**Seaweeds**	***Porphyra* spp.**				***S. polyschides***				***Ulva* spp.**			
**Harvest**	Summer		Autumn		Summer		Autumn		Summer		Autumn	
**Fatty acids**	%	SD	%	SD	%	SD	%	SD	%	SD	%	SD
**SFA**												
C14:0	3.8E-02	6.8E-03	4.2E-02	1.3E-02	3.8E-02	6.2E-03	3.4E-02	6.5E-04	3.4E-02	6.2E-03		
C15:0							3.7E-03	1.4E-04				
C16:0	2.4E-01	1.7E-02	2.6E-01	3.2E-02	3.3E-01	8.8E-03	2.6E-01	1.7E-03	3.6E-01	2.0E-02	3.0E-01	9.3E-03
C17:0							9.4E-03	3.5E-04				
C18:0	1.4E-01	7.3E-03	1.4E-01	5.4E-02	7.1E-02	1.9E-02	4.4E-02	4.3E-04	1.9E-01	2.3E-02	1.8E-01	1.4E-02
C20:0							1.0E-02	8.9E-05				
C21:0												
C22:0	3.3E-02	5.7E-03					7.3E-03	1.9E-04				
C23:0												
C24:0												
**MUFA**												
C14:1 n-5	2.0E-02	3.4E-03										
C15:1 n-5												
C16:1 n-7	5.1E-02	7.7E-03					2.7E-02	8.0E-04			4.9E-02	6.6E-03
C17:1 n-7	2.5E-02	4.0E-03										
C18:1 n-9 t												
C18:1 n-9 c	9.1E-02	9.1E-03	9.9E-02	1.4E-03	1.9E-01	3.3E-03	1.3E-01	4.0E-03	1.2E-01	1.7E-03	1.1E-01	6.0E-03
C20:1n-9	3.7E-02	1.4E-03										
C22:1 n-9	1.1E-01	1.9E-03	1.6E-01	1.7E-03	2.1E-01	5.5E-03	1.8E-01	9.5E-04	2.7E-02	3.0E-03		
C24:1 n-9												
**PUFA**												
C18:2 n-6 t												
C18:2 n-6 c	1.8E-02	1.3E-03	4.3E-02	1.1E-02	6.3E-02	1.9E-03	1.5E-01	3.4E-03	6.6E-02	1.5E-02	7.1E-02	2.8E-03

C18:3 n-3					5.5E-02	5.8E-03	4.7E-02	8.8E-04	1.4E-01	1.8E-02	2.8E-01	2.0E-02
C18:3 n-6							3.3E-02	6.5E-04				
C20:2 n-6							2.2E-03	2.2E-04				
C20:3 n-3							7.2E-04	7.9E-05				
C20:3 n-6							1.0E-03	1.1E-04				
C20:4 n-6												
C20:5 n-3	2.0E-01	6.4E-03	2.5E-01	1.8E-02	4.8E-02	4.9E-03	5.5E-02	2.7E-03	6.1E-02	5.1E-03		
C22:2 n-6												
C22:6 n-3												
**ΣSFA**	4.5E-01		4.5E-01		4.4E-01		3.7E-01		5.9E-01		4.8E-01	
**ΣMUFA**	3.4E-01		2.6E-01		4.0E-01		3.4E-01		1.5E-01		1.6E-01	
**ΣPUFA**	2.1E-01		2.9E-01		1.7E-01		2.9E-01		2.6E-01		3.5E-01	
**Σω3**	2.0E-01		2.5E-01		1.0E-01		1.0E-01		2.0E-01		2.8E-01	
**Σω6**	1.8E-02		4.3E-02		6.3E-02		1.8E-01		6.6E-02		7.1E-02	
**AI**	7.1E-01		7.6E-01		8.5E-01		6.4E-01		1.2E+00		5.9E-01	
**TI**	4.8E-01		4.6E-01		7.9E-01		5.9E-01		7.9E-01		4.9E-01	
**HH**	1.1E+00		1.3E+00		9.7E-01		1.3E+00		1.0E+00		1.5E+00	

Table S2. Experimental values for the lipid profile and quality indices of the commercial seaweeds.

**Seaweeds**	***A. nodosum***		***C. crispus***				***F. vesiculosus***		***Laminaria* spp.**		***Porphyra* spp.**		***Ulva* spp.**		***U. pinnatifida***	
**Commercial production**	Wild		Wild		Aquaculture		Wild		Wild		Aquaculture		Aquaculture		Wild	
**Fatty acids**	%	SD	%	SD	%	SD	%	SD	%	SD	%	SD	%	SD	%	SD
**SFA**																
C12:0	6.2E-04	4.9E-05														
C13:0																
C14:0	9.0E-02	2.0E-03	2.6E-02	6.3E-03	4.7E-02	8.0E-04	1.2E-01	6.4E-03	3.7E-02	3.4E-03	2.0E-02	1.5E-03	3.3E-02	6.2E-04	3.2E-02	1.6E-03
C15:0	1.9E-03	1.3E-04	2.7E-03	7.7E-05	4.5E-03	1.7E-04	3.7E-03	5.3E-05	2.2E-03	2.3E-04			3.2E-03	2.5E-04		
C16:0	9.7E-02	5.4E-03	2.3E-01	2.0E-02	2.4E-01	1.1E-02	1.4E-01	2.0E-03	2.3E-01	1.7E-02	2.3E-01	1.4E-02	2.1E-01	9.3E-04	2.4E-01	1.2E-02
C17:0	1.7E-03	5.7E-05	2.2E-03	3.0E-04			5.9E-03	4.2E-04	6.1E-03	2.3E-04			2.2E-03	1.1E-04	9.1E-03	1.8E-04
C18:0	9.7E-03	9.2E-04	7.0E-02	4.2E-03	9.4E-02	6.3E-03	1.5E-02	4.3E-04	3.5E-02	1.4E-04	9.1E-02	2.6E-03	6.3E-02	7.1E-05	7.7E-02	4.1E-03
C20:0	4.5E-03	2.8E-04	1.6E-03	7.1E-05			4.0E-03	2.9E-04	3.4E-03	5.6E-05			2.1E-03	1.1E-04	1.3E-02	2.2E-04
C21:0	5.2E-04	7.0E-06														
C22:0	8.0E-03	1.9E-04					4.6E-03	1.5E-04					3.5E-03	9.8E-06		
C23:0	8.3E-03	3.4E-04					1.7E-03	3.4E-05								
C24:0	1.9E-03	6.7E-05	1.4E-03	1.1E-04			2.2E-03	9.5E-05					1.3E-02	1.1E-03		
**MUFA**																
C14:1 n-5	2.4E-03	1.6E-04					1.2E-03	8.6E-05								
C15:1 n-5																
C16:1 n-7	1.5E-02	1.3E-03	3.3E-02	8.0E-03	6.8E-02	1.1E-03	2.5E-02	4.7E-04	3.9E-02	2.6E-03	3.9E-02	3.3E-03	4.8E-02	5.7E-05	9.6E-03	5.6E-04
C17:1 n-7	9.1E-04	2.3E-05					1.1E-03	4.2E-05					1.3E-03	5.4E-05		
C18:1 n-9 t			3.6E-03	7.9E-05									3.9E-03	1.2E-04		
C18:1 n-9 c	4.0E-01	8.7E-03	2.9E-01	2.1E-02	2.4E-01	1.4E-03	2.4E-01	6.9E-03	2.1E-01	1.9E-02	3.0E-01	5.5E-03	2.9E-01	7.3E-04	2.0E-01	1.4E-03
C20:1n-9	2.5E-03	1.7E-04	2.0E-02	5.6E-03	7.4E-02	6.5E-03	1.2E-02	5.8E-04	1.4E-02	1.3E-03	2.3E-02	4.4E-04	4.7E-02	6.4E-04		
C22:1 n-9	1.4E-01	9.4E-03	1.1E-02	1.4E-04	1.5E-02	2.1E-04	1.3E-01	4.7E-03	1.5E-01	1.8E-02	1.6E-02	1.8E-04	1.6E-02	9.1E-05	1.1E-01	1.9E-03
C24:1 n-9	3.4E-04	3.0E-05	2.7E-03	1.8E-04			9.0E-04	7.3E-05					4.5E-03	5.6E-05		
**PUFA**																
C18:2 n-6 t																
C18:2 n-6 c	1.0E-01	6.1E-04	1.2E-01	1.3E-02	8.5E-02	5.9E-03	9.9E-02	1.2E-04	1.1E-01	9.5E-03	1.4E-01	1.0E-02	1.1E-01	1.9E-04	1.2E-01	2.4E-03
C18:3 n-3	3.4E-02	1.5E-03	1.2E-01	9.4E-03	3.4E-02	1.9E-03	7.6E-02	2.8E-03	8.1E-02	1.8E-02	9.0E-02	3.3E-03	8.3E-02	1.7E-04	1.3E-01	8.6E-03
C18:3 n-6	1.8E-03	2.3E-05	3.2E-03	1.4E-04			2.3E-03	1.4E-04	3.1E-03	2.1E-04			2.1E-03	2.9E-05		
C20:2 n-6	2.1E-02	3.2E-04	3.1E-03	6.5E-05			1.2E-02	3.0E-04	2.1E-03	1.9E-04			3.6E-03	9.2E-06		
C20:3 n-3	3.3E-03	1.0E-04					1.7E-03	6.6E-05								
C20:3 n-6	9.2E-04	7.1E-05					9.9E-04	4.5E-05					1.7E-03	2.4E-05		

C20:4 n-6					4.0E-03	5.9E-05	3.5E-04	3.1E-05	9.6E-04	2.2E-05			3.5E-03	2.1E-04		
C20:5 n-3	4.5E-02	3.8E-03	2.2E-02	4.8E-03	4.6E-02	3.2E-03	8.7E-02	1.6E-03	5.7E-02	5.6E-03	1.9E-02	8.9E-05	1.7E-02	7.8E-04	4.3E-02	3.0E-03
C22:2 n-6																
C22:6 n-3	2.0E-03	2.7E-04	3.8E-02	2.2E-03	5.5E-02	4.3E-03	8.8E-03	1.0E-04	2.3E-02	1.4E-03	2.7E-02	8.3E-06	3.5E-02	1.6E-03	1.1E-02	2.7E-04
**ΣSFA**	2.2E-01		3.3E-01		3.8E-01		3.0E-01		3.1E-01		3.4E-01		3.3E-01		3.7E-01	
**ΣMUFA**	5.7E-01		3.6E-01		3.9E-01		4.1E-01		4.2E-01		3.8E-01		4.1E-01		3.2E-01	
**ΣPUFA**	2.1E-01		3.1E-01		2.2E-01		2.9E-01		2.7E-01		2.8E-01		2.6E-01		3.0E-01	
**Σω3**	8.4E-02		1.8E-01		1.3E-01		1.7E-01		1.6E-01		1.4E-01		1.4E-01		1.8E-01	
**Σω6**	1.3E-01		1.3E-01		8.9E-02		1.1E-01		1.1E-01		1.4E-01		1.2E-01		1.2E-01	
**AI**	5.9E-01		4.9E-01		6.9E-01		8.7E-01		5.4E-01		4.7E-01		5.1E-01		5.9E-01	
**TI**	1.6E-01		1.5E-01		1.7E-01		1.2E-01		1.4E-01		2.1E-01		1.7E-01		1.6E-01	
**HH**	3.1E+00		2.3E+00		1.6E+00		2.0E+00		1.8E+00		2.3E+00		2.2E+00		1.8E+00	

Table S3. Concentrations of aliphatic hydrocarbons (μg/g dry mass) and respective deviations found in seaweeds under study.

Species	Seaweeds collected	C18	C19	C20	C22	C24	C28	C32	C36	∑AHCs
A. nodosum	Su	12 ± 1	5.7 ± 0.7	2.3 ± 0.2	1.1 ± 0.1	0.68 ± 0.09	0.19 ± 0.03	0.34 ± 0.13	0.83 ± 0.01	31 ± 12
	Wi	n.d.	0.63 ± 0.03	0.67 ± 0.04	0.35 ± 0.04	5.9 ± 0.1	0.42 ± 0.01	0.25 ± 0.01	n.d.	11 ± 5
C. crispus	Sp	1.9 ± 0.2	0.87 ± 0.09	0.60 ± 0.07	0.24 ± 0.07	0.24 ± 0.03	0.085 ± 0.017	n.d.	n.d.	5.4 ± 2.2
	Su	1.2 ± 0.2	0.61 ± 0.05	0.46 ± 0.09	0.31 ± 0.10	0.30 ± 0.06	0.22 ± 0.03	0.24 ± 0.05	n.d.	4.6 ± 2.0
	Au	2.7 ± 0.3	0.31 ± 0.01	0.59 ± 0.05	0.46 ± 0.10	0.20 ± 0.03	0.20 ± 0.04	n.d.	n.d.	6.1 ± 2.5
	Cm[a]	11 ± 1	0.20 ± 0.01	0.24 ± 0.02	0.22 ± 0.01	1.1 ± 0.3	3.3 ± 0.9	1.2 ± 0.4	0.23 ± 0.09	13 ± 10
	Cm[b]	0.27 ± 0.02	0.32 ± 0.04	0.41 ± 0.01	0.24 ± 0.02	0.17 ± 0.02	0.45 ± 0.03	0.076 ± 0.010	0.21 ± 0.01	2.8 ± 1.1
F. spiralis	Sp	4.4 ± 0.2	1.8 ± 0.2	1.1 ± 0.1	0.52 ± 0.03	0.34 ± 0.12	0.45 ± 0.04	n.d.	n.d.	11 ± 5
	Su	4.7 ± 0.6	2.0 ± 0.3	1.2 ± 0.2	0.71 ± 0.14	0.36 ± 0.05	0.50 ± 0.10	n.d.	n.d.	13 ± 6
F. vesiculosus	Cm[b]	0.77 ± 0.11	1.2 ± 0.1	0.78 ± 0.01	0.75 ± 0.03	2.2 ± 0.09	0.73 ± 0.02	0.50 ± 0.13	2.8 ± 0.1	12 ± 4
Gracilaria spp.	Sp	1.0 ± 0.1	0.64 ± 0.04	0.19 ± 0.01	0.26 ± 0.04	0.26 ± 0.01	0.27 ± 0.01	n.d.	n.d.	3.6 ± 1.5
	Au	3.2 ± 0.2	0.41 ± 0.02	0.48 ± 0.05	0.68 ± 0.05	0.20 ± 0.04	0.24 ± 0.07	n.d.	n.d.	7.0 ± 2.9
L. ochroleuca	Sp	0.58 ± 0.11	0.56 ± 0.10	0.26 ± 0.01	0.25 ± 0.08	0.21 ± 0.04	0.19 ± 0.04	0.14 ± 0.04	n.d.	3.1 ± 1.3
	Su	n.d.	0.16 ± 0.01	0.28 ± 0.06	0.18 ± 0.01	0.41 ± 0.08	0.31 ± 0.04	0.067 ± 0.021	0.49 ± 0.01	2.4 ± 0.8
Laminaria spp.	Cm[b]	n.d.	1.1 ± 0.1	0.57 ± 0.03	0.60 ± 0.06	3.4 ± 0.2	0.387568	0.092 ± 0.03	n.d.	8.3 ± 3.5
O. pinnatifida	Sp	7.1 ± 0.6	2.6 ± 0.3	1.3 ± 0.1	0.72 ± 0.08	0.72 ± 0.21	0.63 ± 0.06	0.41 ± 0.06	0.34 ± 0.13	19 ± 8
	Au	n.d.	0.17 ± 0.03	0.17 ± 0.04	0.072 ± 0.010	0.036 ± 0.007	0.24 ± 0.04	0.039 ± 0.009	n.d.	1.0 ± 0.4
Porphyra spp.	Su	2.8 ± 0.2	1.2 ± 0.1	0.59 ± 0.10	0.54 ± 0.09	0.57 ± 0.11	0.44 ± 0.08	0.82 ± 0.18	n.d.	9.6 ± 4.0
	Au	7.4 ± 0.5	1.0 ± 0.2	1.2 ± 0.1	1.6 ± 0.3	0.45 ± 0.12	0.38 ± 0.05	n.d.	n.d.	17 ± 7
	Cm[b]	0.56 ± 0.02	0.52 ± 0.05	0.58 ± 0.05	0.33 ± 0.01	0.044 ± 0.012	0.47 ± 0.02	0.085 ± 0.009	0.52 ± 0.10	4.1 ± 1.5
Ulva spp.	Su	3.2 ± 0.2	3.7 ± 0.4	1.1 ± 0.1	0.62 ± 0.01	0.32 ± 0.01	0.49 ± 0.13	n.d.	n.d.	13 ± 5
	Au	6.3 ± 0.8	1.5 ± 0.1	1.8 ± 0.2	1.1 ± 0.1	0.19 ± 0.03	0.22 ± 0.02	n.d.	n.d.	15 ± 6
	Cm[b]	0.60 ± 0.03	0.46 ± 0.04	0.51 ± 0.02	0.55 ± 0.06	4.0 ± 0.1	7.7 ± 0.4	1.9 ± 0.09	n.d.	21 ± 9

	Su	10 ± 1	5.3 ± 0.7	2.2 ± 0.5	1.1 ± 0.4	0.72 ± 0.16	0.48 ± 0.14	0.29 ± 0.07	n.d.	28 ± 12
U. pinnatifida	Wi	2.3 ± 0.1	0.56 ± 0.04	0.45 ± 0.02	0.45 ± 0.03	0.14 ± 0.01	0.12 ± 0.01	n.d.	n.d.	5.4 ± 2.3
	Cm[a]	0.20 ± 0.03	0.25 ± 0.06	0.51 ± 0.07	0.28 ± 0.02	3.0 ± 0.9	1.9 ± 0.2	0.83 ± 0.22	0.67 ± 0.05	10 ± 4

Sp: Spring; Su: Summer; Au; Autumn; Wi: Winter; Cm[a]: Commercial wild origin; Cm[b]: Commercial aquaculture origin.

The seaweeds harvested at different times of the year and the ones acquired in Portugal were analyzed. It should be noted that table S3 does not show the results for all samples since even after adding activated carbon, it was impossible to remove the color from the sample altogether, and it was not possible to proceed with the analysis of the sample by GC.
